# Supplementary material for: MetaRibo-Seq measures translation in microbiomes
Source: Nat Commun. 2020 Jun 29;11:3268. doi: 10.1038/s41467-020-17081-z (PMC7324362; doi:10.1038/s41467-020-17081-z)
Supplement: Supplementary file 10 — Supplementary Data 7 [file 41467_2020_17081_MOESM10_ESM.zip › File2/Confidence_High_Taxonomy/438203_out.krona.html]

Javascript must be enabled to view this page.

members
magnitude
magnitudeUnassigned
count
unassigned
taxon
rank

438203\_out

14

14
superkingdom
2

14
1239
phylum

14
186801
class

14
order
186802

14
186803
family

14
841
genus

14

SRS013940\_contig\_number\_16408SRS014287\_contig\_number\_15836SRS014948\_contig\_number\_10373SRS015190\_contig\_number\_15302SRS015264\_contig\_number\_21815SRS022137\_contig\_number\_contig-100\_346.110666SRS024549\_contig\_number\_4200SRS043411\_contig\_number\_3696SRS050422\_contig\_number\_36955SRS056273\_contig\_number\_18167SRS101433\_contig\_number\_1039SRS1041130\_contig\_number\_6015SRS149879\_contig\_number\_18671SRS893380\_contig\_number\_contig-100\_1155.1156
166486
species
